# Single nucleotide polymorphisms in DNA repair genes as risk factors associated to prostate cancer progression

**DOI:** 10.1186/s12881-014-0143-0

**Published:** 2014-12-24

**Authors:** Luis Alberto Henríquez-Hernández, Almudena Valenciano, Palmira Foro-Arnalot, María Jesús Álvarez-Cubero, José Manuel Cozar, José Francisco Suárez-Novo, Manel Castells-Esteve, Pablo Fernández-Gonzalo, Belén De-Paula-Carranza, Montse Ferrer, Ferrán Guedea, Gemma Sancho-Pardo, Jordi Craven-Bartle, María José Ortiz-Gordillo, Patricia Cabrera-Roldán, Estefanía Herrera-Ramos, Carlos Rodríguez-Gallego, Juan Ignacio Rodríguez-Melcón, Pedro C Lara

**Affiliations:** Radiation Oncology Department, Hospital Universitario de Gran Canaria Dr. Negrín, C/Barranco de La Ballena s/n, CP 35010 Las Palmas de Gran Canaria, Las Palmas Spain; Instituto Canario de Investigación del Cáncer, Las Palmas, Spain; Clinical Sciences Department, Universidad de Las Palmas de Gran Canaria, Las Palmas, Spain; Institud d’Oncologia Radioteràpica, Hospital de la Esperanza. Parc de Salut Mar, Universidad Pompeu Fabra, Barcelona, Spain; Laboratory of Genetic Identification, Legal Medicine and Toxicology Department, Facultad de Medicina, Universidad de Granada, Granada, Spain; GENYO, Pfizer-University of Granada-Andalusian Government Centre for Genomics and Oncological Research, Granada, Spain; Department of Urology, Hospital Universitario Virgen de las Nieves, Granada, Spain; Department of Urology, Hospital Universitari de Bellvitge, L’Hospitalet de Llobregat, Spain; Radiation Oncology Department, Onkologikoa, Guipuzcoa Spain; Health Services Research Group, Institut de Recerca Hospital del Mar (IMIM), Barcelona, Spain; Department of Radiation Oncology, Institut Català d’Oncologia (ICO), L’Hospitalet de Llobregat, Spain; Radiation Oncology Department, Hospital de la Santa Creu i Sant Pau, Barcelona, Spain; Radiation Oncology Department, Hospital Universitario Virgen del Rocío, Sevilla, Spain; Department of Immunology, Hospital Universitario de Gran Canaria Dr. Negrín, Las Palmas, Spain; Department of Medical and Surgical Sciences, Universidad de Las Palmas de Gran Canaria, Las Palmas, Spain

**Keywords:** Single nucleotide polymorphism, *ERCC1*, *ATM*, Prostate cancer, OpenArray, DNA repair, Spanish cohort

## Abstract

**Background:**

Besides serum levels of PSA, there is a lack of prostate cancer specific biomarkers. It is need to develop new biological markers associated with the tumor behavior which would be valuable to better individualize treatment. The aim of this study was to elucidate the relationship between single nucleotide polymorphisms (SNPs) in genes involved in DNA repair and prostate cancer progression.

**Methods:**

A total of 494 prostate cancer patients from a Spanish multicenter study were genotyped for 10 SNPs in *XRCC1, ERCC2, ERCC1, LIG4, ATM* and *TP53* genes. The SNP genotyping was made in a Biotrove OpenArray® NT Cycler. Clinical tumor stage, diagnostic PSA serum levels, and Gleason score at diagnosis were obtained for all participants. Genotypic and allelic frequencies were determined using the web-based environment SNPator.

**Results:**

SNPs rs11615 (*ERCC1*) and rs17503908 (*ATM*) appeared as risk factors for prostate cancer aggressiveness. Patients wild homozygous for these SNPs (AA and TT, respectively) were at higher risk for developing cT2b – cT4 (OR = 2.21 (confidence interval (CI) 95% 1.47 – 3.31), p < 0.001) and Gleason scores ≥ 7 (OR = 2.22 (CI 95% 1.38 – 3.57), p < 0.001), respectively. Moreover, those patients wild homozygous for both SNPs had the greatest risk of presenting D’Amico high-risk tumors (OR = 2.57 (CI 95% 1.28 – 5.16)).

**Conclusions:**

Genetic variants at DNA repair genes are associated with prostate cancer progression, and would be taken into account when assessing the malignancy of prostate cancer.

**Electronic supplementary material:**

The online version of this article (doi:10.1186/s12881-014-0143-0) contains supplementary material, which is available to authorized users.

## Background

Prostate cancer (PCa) is a complex disease highly influenced by hormonal and genetic factors which would condition the tumor behavior. Tumor staging, tumor grading in terms of Gleason score and diagnostic prostate specific antigen (PSA) serum levels are clinically used to classify patients into different prognostic risk groups which will condition treatment decisions. However, it is estimated that 293 men have to be screened and 12 men have to be treated to avoid one death related to PCa. Although recent advances in genomic research have made possible to identify new biomarkers for PCa, results are inconclusive [1] and it seems to be a need for new biomarkers of tumor behavior.

DNA is constantly damaged by endogenous oxygen free radicals and exogenous chemicals; thus, different repair pathways are available to reverse the different types of DNA damage [2]. Defects in these DNA repair pathways may increase persistent mutations in daughter cell generations, genomic instability, and ultimately a more aggressive disease [3]. DNA repair genes play a major role in the DNA mismatch repair pathway, which includes base excision repair (BER), nucleotide excision repair (NER), mismatch repair (MMR) and double strand break repair (DSBR); and are essential for maintaining the integrity of the genome [4]. Genetic variations in genes involved in DNA repair would confer susceptibility to the tumor, and would be associated to disease aggressiveness through the alteration of DNA repair pathways [5], which could induce tumor transformation and acquisition of oncologic properties. Single nucleotide polymorphisms (SNPs) are defined as inherited mutations that are present in more than 1% of the population. Given that there are millions of SNPs in the entire human genome, a major difficulty is to choose target SNPs that are most likely to affect phenotypic functions and ultimately contribute to disease development. Candidate gene studies are focused on the selection of genes that have been previously related to a disease, and thus come with prior knowledge about gene function. Among dozens of genes directly involved in DNA repair in humans [6], six of them have been previously studied in a wide series of Spanish PCa patients based on its relevance in the mechanism of the disease [7]: X-ray repair cross-complementing protein 1 (*XRCC1*), excision repair cross-complementing rodent repair deficiency, complementation group 2 (*ERCC2*), excision repair cross-complementing rodent repair deficiency, complementation group 1 (*ERCC1*), ligase IV (*LIG4*), ataxia telangiectasia mutated (*ATM*), and tumor protein p53 (*TP53*).

The ethnic origin of the studied population is a key factor in gene-association studies. In that sense, the literature is full of genetic variances that are risk factors for certain diseases among subjects of an ethnic origin but are not valid among subjects from other ethnicities [8,9]. Moreover, we have previously reported that differences in distribution of genotypes within different populations of the same ethnicity could be an important confounding factor in gene-association studies [7]. In that sense, since cohorts are often multi-ethnic, the STROGAR guidelines recommend to report whether ethnicity was controlled for in reporting of genotype–phenotype association, and encourage the use of cohorts from ethnically uniform populations [10].

We hypothesize here that genetic variations in DNA repair genes would confer different behavior to PCa cells and would result in a distinct clinical phenotype. Thus, the aim of the present study was to elucidate the relationship between 10 SNPs located in 6 different genes involved in DNA repair that have been classically associated to PCa risk [3], and tumor aggressiveness in a wide set of Spanish PCa patients.

## Methods

### Patients

A total of 601 patients with non-metastatic localized prostate cancer (PCa) from 4 different regions of Spain (15.14% from Andalusia, 8.48% from Basque Country, 39.60% from Canary Islands and 36.77% from Catalonia) were included in the study [7]. We have previously shown that differences in the distribution of genotypes within different populations of the same ethnicity are an important confounding factor in genetics epidemiology. In that sense, Andalusian subjects showed the greatest differences [7]. Thus, to homogenize the sample and minimize bias, we excluded this subset of patients from the analyses. A total of 494 PCa were included in the present study. All patients provided written informed consent before sample collection. The study was approved by the Research and Ethics Committee of each institution participant in the study: Hospital Universitario de Gran Canaria Dr. Negrín (Las Palmas de Gran Canaria), Hospital de la Esperanza - Parc de Salut Mar (Barcelona), Hospital Universitario Virgen de las Nieves (Granada), Hospital Universitari de Bellvite (L'Hospitalet de Llobregat), Onkologikoa (Guipuzcoa), Institut Català d'Oncologia (L'Hospitalet de Llobregat), Hospital de la Santa Creu i Sant Pau (Barcelona) and Hospital Universitario Virgen del Rocío (Sevilla).

Clinical tumor size (cT), diagnostic PSA serum levels, and Gleason score [11] were recruited for all PCa patients. Clinical tumor size was assessed by digital rectal examination (DRE) followed by transrectal ultrasonography (TRUS) and magnetic resonance imaging (MRI); PSA serum levels were assessed by chemiluminescence in an Architect i2000 analyzer (Abbott Laboratories, IL, USA); Gleason score was determined in the biopsy specimen by a pathologist. Subjects were categorized into three risk-based recurrence groups according to D’Amico classification [12]: low, intermediate, and high risk. After collecting demographic and clinical data, a blood sample was taken after the signature of informed consent. All samples were sent by courier to the Hospital Universitario de Gran Canaria, for DNA isolation and genotype analyses as follows.

### DNA isolation and quantification

DNA was obtained from blood samples at the Hospital Universitario de Gran Canaria Dr. Negrín. DNA was isolated from 300 μl of whole-blood in an iPrep™ Purification Instrument using the iPrep™ PureLink™ gDNA Blood Kit (Invitrogen, by Life Technologies, Carlsbad, CA), and its integrity was determined by NanoDrop ND-1000 (NanoDrop Technologies, Wilmington, DE).

### Genes and SNPs

A total of 10 SNPs in 6 different genes involved in DNA repair were studied (Table 1): *XRCC1* (involved in base excision repair [6]), rs25487, rs25489, rs1799782; *ERCC2* (involved in nucleotide excision repair [6]), rs13181; *ERCC1* (involved in nucleotide excision repair [6]), rs11615; *LIG4* (involved in double-strand break repair [6]), rs1805388, rs1805386; *ATM* (involved in double-strand break repair [6]), rs17503908, rs1800057; and *TP53* (involved in double-strand break repair [6]), rs1042522.

**Table 1:** **Description of clinical variables**

**Clinical**	**n**	**(%)**
Clinical tumor size (cT)		
cT1a – cT2a	270	(54.7)
cT2b – cT2c	141	(25.8)
cT3 – cT4	66	(13.4)
NA	17	(3.4)
Initial PSA (ng/mL)		
<10	306	(61.9)
10 – 19.99	103	(20.9)
>20	79	(16.0)
NA	6	(1.2)
Gleason score		
<7	226	(45.7)
7	195	(39.5)
>7	71	(14.4)
NA	2	(0.4)
D’Amico group		
Low	120	(24.3)
Intermediate	184	(37.2)
High	173	(35.0)
NA	17	(3.4)

*Abbreviations*: *PSA* prostate specific antigen, *NA* not available.

### Genotyping

The SNP genotyping was performed in a Biotrove OpenArray® NT Cycler (Applied Biosystems, Foster City, CA) [13]. DNA samples loaded in the OpenArray (OA) had a A260/A280 and A260/230 ratios of 1.7-1.9, and were adjusted to 50 ng/μl. A total of 300 ng of genomic DNA was used. A final amount of 150 ng was incorporated into the array with the autoloader, and was genotyped according to the manufacturer's recommendations. A non-template control (NTC) consisting of DNase-free double-distilled water was introduced within each assay. When the DNA and master mix were transferred, the loaded OA plate was filled with an immersion fluid and sealed with glue. The multiplex TaqMan assay reactions were carried out in a Dual Flat Block (384-well) GeneAmp PCR System 9700 (Applied Biosystems) with the following PCR cycle: an initial step at 93°C for 10 minutes followed by 50 cycles of 45 seconds at 95°C, 13 seconds at 94°C and 2:14 minutes at 53°C; followed by a final step during 2 minutes at 25°C and holding at 4°C.

The fluorescence was read using the OpenArray® SNP Genotyping Analysis software version 1.0.5. (Applied Biosystems). The genotyping analysis was made with the TaqMan Genotyper software version 1.0.1. (available at: http://www.invitrogen.com) using autocalling as the call method. The quality value of the data points was determined by a threshold above 0.95. Genotype analysis was performed with the same batch of chips and by the same investigator, as previously reported [7].

### Statistical analysis

Genotype and allelic frequencies were determined using the web-based environment SNPator (SNP Analysis To Results, from the Spain's National Genotyping Centre and the National Institute for Bioinformatics) [14]. Relative excess of heterozygosity was determined to check compatibility of genotype frequencies with Hardy-Weinberg equilibrium (HWE). Thus, p-values from the standard exact HWE lack of fit test were calculated using SNPator.

Comparisons of genotypic and allelic frequencies were also done in SNPator.

All additional statistical analyses were performed using PASW Statistics 15 (IBM Corporation, Armonk, NY, USA).

## Results

The majority of PCa patients were cT1a – cT2a (54.7%), PSA < 10 ng/mL (61.9%), and Gleason score < 7 (45.7%). Subsequently, a total of 120 patients (24.3%) were classified as low risk tumors according to D’Amico classification. We did not observed clinical differences among different regions of Spain (data not shown). Distribution of clinical variables is detailed in Table 1.

All the genotyped samples met the quality criteria stated above. A total of 494 PCa patients were genotyped for 10 SNPs. Of the 4,940 possible determinations, 97.17% were successfully genotyped. The genotypic and allelic frequencies are shown in Table 2. Minor allele frequencies (MAF) were similar to those reported in the literature. All SNPs were in HWE.

**Table 2:** **Genotypic and allelic frequencies among Spanish prostate cancer patients**

**Gene/SNP**	**Function**	**chr**	**n**	**Genotypic frequencies**						**Allelic frequencies**				**MAF#**	**Functional consequence#**
***XRCC1***	BER	19q13													
rs25487*			436	CC	0.42	CT	0.48	TT	0.10	C	0.66	T	0.34	0.26	Missense
rs25489			483	CC	0.88	CT	0.12	TT	0.00	C	0.94	T	0.06	0.06	Missense
rs1799782*			487	AA	0.00	AG	0.12	GG	0.88	A	0.06	G	0.94	0.13	Missense
***ERCC2***	NER	19q13													
rs13181			482	GG	0.10	GT	0.47	TT	0.43	G	0.34	T	0.66	0.24	Missense
***ERCC1***	NER	19q13													
rs11615			488	AA	0.39	AG	0.46	GG	0.16	A	0.61	G	0.39	0.36	Synonymous codon
***LIG4***	DSBR	13q23													
rs1805388^†^			488	AA	0.04	AG	0.25	GG	0.71	A	0.17	G	0.83	0.15	Missense
rs1805386^†^			480	AA	0.70	AG	0.26	GG	0.04	A	0.83	G	0.17	0.10	Synonymous codon
***ATM***	DSBR	11q22													
rs17503908			486	GG	0.00	GT	0.18	TT	0.81	G	0.09	T	0.91	0.06	Intron variant
rs1800057			486	CC	0.94	CG	0.06	GG	0.00	C	0.97	G	0.03	0.02	Missense
***TP53***	DSBR	17p13													
rs1042522			484	CC	0.59	CG	0.35	GG	0.06	C	0.76	G	0.24	0.39	Missense

*Abbreviations*: *BER* base excision repair, *NER* nucleotide excision repair, *DSBR* double-strand break repair, *chr* chromosome, *MAF* minor allele frequency.

#Information available at: http://www.ncbi.nlm.nih.gov/projects/SNP/.

*SNPs in perfect linkage disequilibrium.

^†^SNPs in perfect linkage disequilibrium.

Among the 10 analyzed SNPs, rs11615 (minor allele frequency (MAF) = 0.39) and rs17503908 (MAF = 0.09), located in *ERCC1* and *ATM* respectively, were significantly different distributed among PCa patients according to the clinical variables (Additional file 1). Thus, rs11615 was significantly associated to the clinical tumor size (χ^2^ test, p = 0.002) while rs17503908 was associated to the Gleason score (χ^2^ test, p = 0.005). Concerning to rs11615, we observed that among the 259 patients diagnosed as cT1a – cT2a, 175 carried the G allele (67.57%). In the other hand, among the 66 patients diagnosed as cT3 – cT4, 31 carried the G allele (46.97%) (Additional file 2). With respect to rs17503908, 169 of the 224 patients (75.45%) scored with Gleason <7 were genotyped as TT, while 59 of the 70 patients (84.29%) scored with Gleason >7 were genotyped as TT (Additional file 2).

We explored the specific role of the SNPs rs11615 and rs17503908 in relation to the associated clinical variables. For this, we conducted the analysis according to various genetic models: recessive, dominant, homozygote, and heterozygote models (Table 3). We observed that patients carrying the GG + AG genotypes for rs16115 were at lower risk for develop cT2b- cT4 tumors than those AA PCa patients (OR = 0.50, (95% Confidence Interval (CI) 0.35 – 0.73), p < 0.0001). This result was also observed in the heterozygote model (Table 3). Similar trend was observed for rs17503908. Those patients carrying the GG + GT genotypes were at lower risk for develop Gleason scores 7 – 10 than those TT PCa patients (OR = 0.48, (95% CI 0.30 – 0.76), p = 0.002), and this trend was also observed in the heterozygote model (Table 3). A and T are the ancestral alleles for rs11615 and rs17503908, respectively. According to our results, wild homozygous genotypes were associated to poor prognosis factors and these AA-rs11615 and TT-rs175803908 PCa patients were at higher risk for develop cT2b – cT4 (OR = 2.21 (CI 95% 1.47 – 3.31), p < 0.001) and Gleason scores ≥ 7 (OR = 2.22 (CI 95% 1.38 – 3.57), p < 0.001), respectively (data not shown).

**Table 3:** **Univariate analysis for polymorphisms rs11615 (** ***ERCC1*** **) and rs17503908 (** ***ATM*** **) and clinical variables**

			**Recessive model**		**Dominant model**		**Homozygote**		**Heterozygote**	
			GG vs. AG + AA		GG + AG vs. AA		GG vs. AA		AG vs. AA	
**Clinical variable**	**n**	**SNP**	OR (95% CI)	P	OR (95% CI)	P	OR (95% CI)	P	OR (95% CI)	P
cT, 1a-2a/2b-4	270/207	rs11615	1.02 (0.62 – 1.68)	0.928	0.50 (0.35 – 0.73)	<0.0001	0.67 (0.39 – 1.16)	0.152	0.45 (0.30 – 0.70)	<0.0001
			GG vs. GT + TT		GG + GT vs. TT		GG vs. TT		GT vs. TT	
			OR (95% CI)	P	OR (95% CI)	P	OR (95% CI)	P	OR (95% CI)	P
Gleason, <7/ ≥ 7	226/266	rs17503908	NA	–	0.48 (0.30 – 0.76)	0.002	NA	–	0.45 (0.28 – 0.72)	0.001

*Abbreviations*: *OR* odds ratio, *CI* confidence interval, *NA* not applicable (due to the limited sample size: only 2 subjects genotyped as GG-rs17503908).

Statistical test: binary logistic regression (Reference category for SNP rs11615: AA. Reference category for SNP rs17503908: TT).

**Figure 1:**
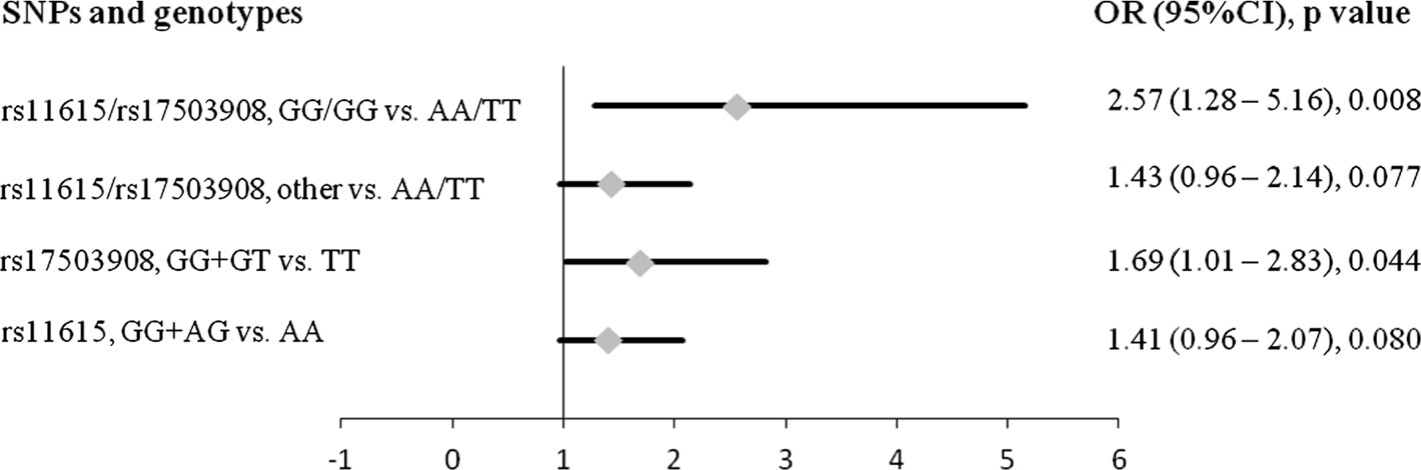
**Forrest plot of odds ratios (OR) with 95% confidence interval (CI) for polymorphisms rs11615 (** ***ERCC1*** **) and rs17503908 (** ***ATM*** **) and D’Amico high risk recurrence group.** Each diamond represents the OR and the horizontal line indicates the 95% CI. For the binary logistic regression, patients were dichotomized in two groups as follows: low – intermediate vs. high D’Amico groups.

We studied the role of rs11615 and rs17503908 in the context of D’Amico risk groups, a classification that estimate the biologic aggressiveness of prostate cancers by grouping them into different risk categories which reflect the risk of cancer growth and spread. Our results showed that those patients carrying the TT genotype for the rs17503908 SNP had an increased risk of developing D’Amico high-risk tumors (OR = 1.69, (CI 95% 1.01 – 2.83), p = 0.044). This trend was not observed for rs11615 SNP (Figure 1). Nonetheless, those PCa patients carrying the AA-TT genotypes for rs16115 and rs17503908 respectively, had the greatest risk of developing D’Amico high-risk tumors (OR = 2.57 (CI 95% 1.28 – 5.16), p = 0.008) (Figure 1). Similar result was obtained when the series was dichotomized in low vs. intermediate-high D’Amico risk groups (OR = 1.98 (CI 95% 1.02 – 3.83), data not shown). These results are in line with previous results showed above, and suggest that these specific genotypes are associated to poor prognosis factors (Additional file 3).

No other polymorphism showed significant associations with clinical variables.

## Discussion

DNA damage occurs very frequently and leads to gene deletions, amplifications, rearrangements, and translocation resulting in the alteration of cell homeostasis and tumor behaviour [2]. In the present study, we investigated the association between 10 SNPs located in DNA repair genes and the aggressiveness of prostate cancer in a wide set of Spanish PCa patients, following a candidate-gene approach based in a previously published study [7]. We observed a strong association of genotypes AA-rs11615 and TT-rs17503908 in the development of clinical variables of worst prognosis. These genetic variations would influence the nucleotide excision repair and double-strand break repair mechanisms of DNA, possible favoring genomic instability and the development of more aggressive cell phenotypes that would cause the appearance of tumors of poor prognosis (i.e. D’Amico high-risk tumors).

There has been an increasing interest on the role of SNPs in the development and progression of PCa. In that sense, SNPs in DNA repair genes have been deeply explored, especially in the prediction of radiation-induced toxicity [15]. *ERCC1* is encoded in chromosome 19q13 and it is involved in nucleotide excision repair, forming with *XPF*, a free, nuclear flap structure-specific endonuclease [6]. In the context of PCa, it has been reported that polymorphisms affecting *ERCC1* may predispose prostate epithelial cells to malignant transformation [16], but there is a lack of information about the role of that gene in disease aggressiveness. Genetic variants at chromosome 19q13 have been evaluated among 7,370 PCa cases, and no association with tumor aggressiveness was observed [1]. Although this study includes a big series, these patients came from different countries, and the ethnic origin was not considered as a confounding factor, especially when differences observed within populations of the same ethnic origin suggest that race is not a sufficient factor to ensure the homogeneity of the sample [17], as we have also previously published [7]. In the present study, we have observed that PCa patients carrying AA-rs11615 genotype were at higher risk for develop bigger tumors. Allele G, in combination with rs3212986 (also located in *ERCC1*), has been associated to low *ERCC1* expression, resulting in reduced DNA repair and better chemotherapy/radiotherapy response [18]. By extension, A allele would be required to maintain normal levels of *ERCC1*, thus conditioning tumor malignancy and response to treatment. In that sense, G allele, which is not the ancestral allele, would confer clinical advantage in terms of tumor size.

*ATM* is encoded in chromosome 11q22. In response to double-strand breaks (DSBs), *ATM* phosphorylates a variety of proteins involved in DSB repair and cell-cycle control [6]. *ATM/ATR* inactivation is a crucial step in promoting androgen-induced genomic instability and prostate carcinogenesis [19], and some missense variants of the *ATM* gene have been shown to confer a moderate increased risk of prostate cancer. Genetic variants at *ATM* have been associated with radiation-induced toxicity [20,21], although none of the previously reported associations were confirmed in a validated study [22]. A similar trend has been observed in the context of tumor aggressiveness [23]. We have observed that PCa patients carrying TT-rs17503908 genotype were at higher risk for develop high-grade tumors. Thus, G allele, which is not the ancestral allele, would confer clinical advantage in terms of Gleason score. Although this is a novel result in PCa, a large variety of distinct *ATM* mutations and variants exist among breast cancer patients, and some of them can contribute to the etiology and progression of the malignancy [24].

Since it seems that wild genotypes represent a risk factor associated with tumor malignancy, we evaluated the combined role of both genotypes in relation to risk groups established by D’Amico. We observed that patients carrying both wild homozygous genotypes (AA + TT) were at higher risk of develop D’Amico high-risk tumors. *ERCC1* and *ATM* are encoded in different chromosomes, thus, there is not conserved combination of SNPs. However, genotypes frequencies for AA-rs11615 and TT-rs17503908 were 0.39 and 0.81, respectively; and a total of 148 PCa patients carried both genotypes (29.9% of the total series). Consequently, a combined analysis would give an idea about the role of both polymorphisms as predictors of tumor malignancy. Nonetheless, it has to be taken into account that functional consequences of rs11615 and rs17503908 are not missense; that is, there is not amino acid substitution in the translated protein. However, cumulative evidence suggest that synonymous mutations are also important, and there is a rapidly growing list of synonymous mutations that lead to human diseases [25,26]. Synonymous SNPs could affect protein function altering RNA secondary structures, affecting RNA stability, and subsequently reducing protein expression [27] and possibly affecting protein folding and function [28]. Therefore, it is possible that both polymorphisms can be important in determining the tumor characteristics of prostate cancer.

Candidate gene association studies are often criticized for their lack of validation. Thus, a replication study using an independent and random cohort of PCa patients should be considered in near future [29]. The present study has some weakness that need to be highlighted: i) although 494 PCa patients seem sufficient to obtain statistically reliable results, it is possible that some results may be of stochastic nature, especially for those SNPs with lower MAF; ii) other factors associated to prostate cancer (i.e. age, familiar aggregation, toxic habits or some kind of diets) have not been taken into account in the present study; iii) the observations should be confirmed in an independent cohort. Despite the above, the present study provides a number of advantages that contribute to their credibility: i) it is a multicenter study that provides patients from different areas of a country, minimizing the bias of studies performed in one hospital; ii) all subjects are from Spanish origin, and it has been reduced the possible influence of intra ethnic variations [7]; iii) all the determinations (4,940 in total) were performed with the same methodology (OpenArray, Applied Biosystems), with the same batch of chips and by the same investigator, thus minimizing biases from technical origin.

## Conclusions

We found that genetic variants at DNA repair genes are associated with clinical variables of poor prognosis for prostate cancer. Prospective studies are required to validate our results.

## Additional files

Additional file 1:
**Significant associations between clinical variables and SNPs.**
Additional file 2:
**Genotype distribution of rs11615 across the different clinical tumor sizes (A) and distribution of rs17503908 across the different Gleason scores (B).**
Additional file 3:
**Combine genotype distribution of SNPs rs11615 and rs17503908 according to the D’ Amico risk -group.** The genotype distribution was statistically different (x^2^test, p = 0.017).
