# Supplementary material for: Single nucleotide polymorphisms in DNA repair genes as risk factors associated to prostate cancer progression
Source: BMC Med Genet. 2014 Dec 24;15:143. doi: 10.1186/s12881-014-0143-0 (PMC4316399; doi:10.1186/s12881-014-0143-0)
Supplement: Additional file 1: — Significant associations between clinical variables and SNPs. [file 12881_2014_143_MOESM1_ESM.doc]

| **Supplementary file 1.** Significant associations between clinical variables and SNPs | | | | | |
| --- | --- | --- | --- | --- | --- |
|  |  | **Clinical tumor size (cT)** | | |  |
| **SNP** | **Genotypes** | cT1a – cT2a | cT2b – cT2c | cT3 – cT4 | **P#** |
| rs11615 | AA | 84 | 63 | 35 | 0.002 |
|  | AG | 140 | 51 | 23 |  |
|  | GG | 35 | 23 | 8 |  |
|  |  | **Gleason score** | | |  |
|  |  | <7 | 7 | >7 |  |
| rs17503908 | GG | 0 | 2 | 0 | 0.005 |
|  | GT | 55 | 22 | 11 |  |
|  | TT | 169 | 166 | 59 |  |
| # Chi square test. | | | | | |
