# Supplementary figures and images for: Single nucleotide polymorphisms in DNA repair genes as risk factors associated to prostate cancer progression
Source: BMC Med Genet. 2014 Dec 24;15:143. doi: 10.1186/s12881-014-0143-0 (PMC4316399; doi:10.1186/s12881-014-0143-0)

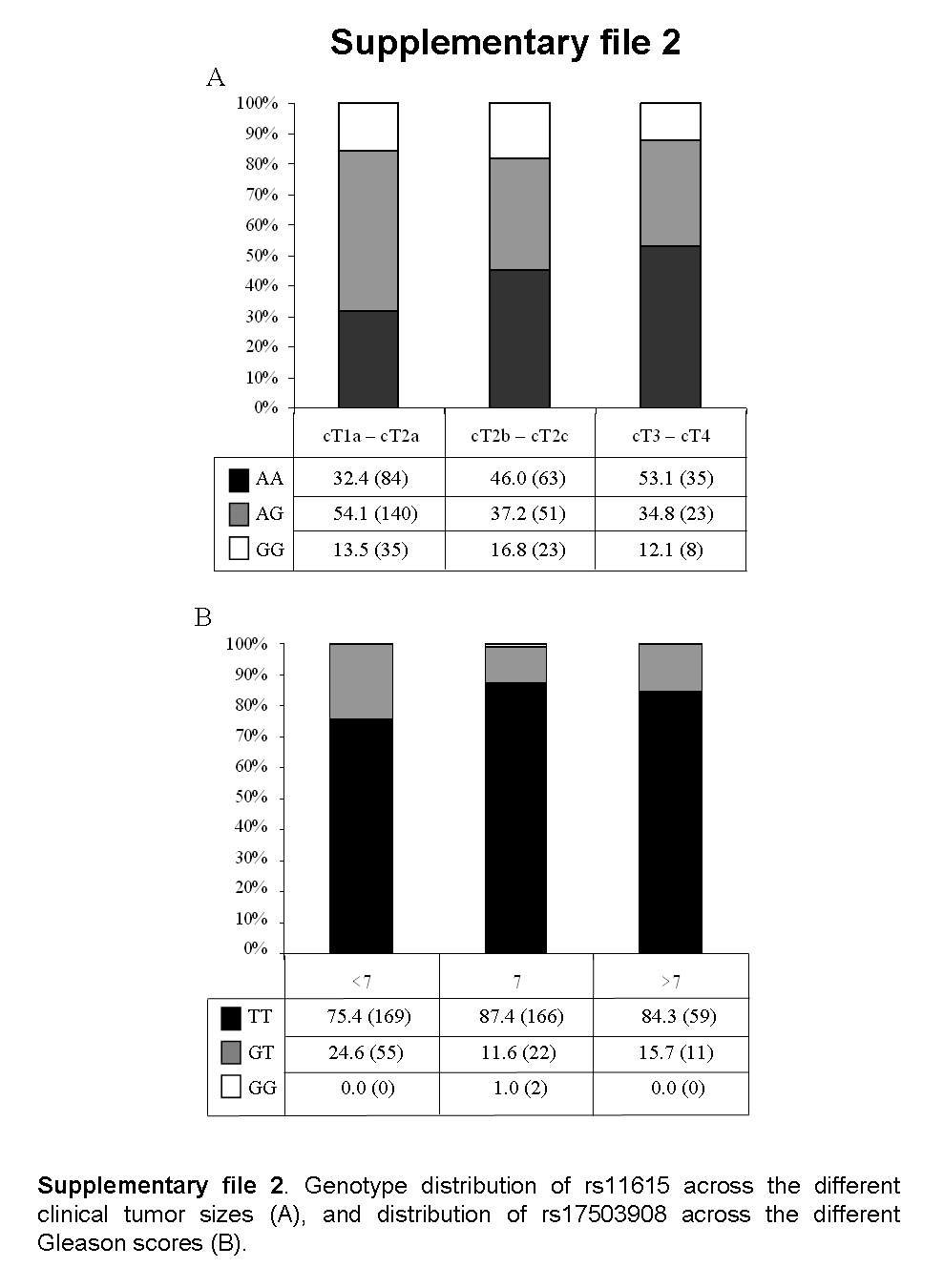

Supplement: Additional file 2: — Genotype distribution of rs11615 across the different clinical tumor sizes (A) and distribution of rs17503908 across the different Gleason scores (B). [file 12881_2014_143_MOESM2_ESM.tiff]

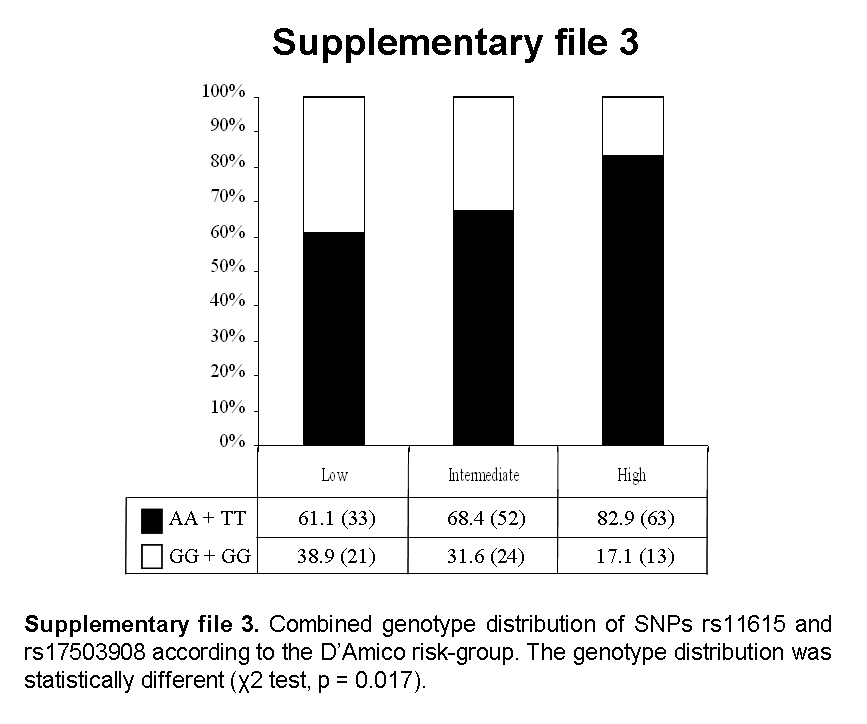

Supplement: Additional file 3: — Combine genotype distribution of SNPs rs11615 and rs17503908 according to the D’ Amico risk -group. The genotype distribution was statistically different (x2test, p = 0.017). [file 12881_2014_143_MOESM3_ESM.tiff]
